# Functional Outcomes of Patients With Sternoclavicular Joint Infection After Extended Resection

**DOI:** 10.1016/j.atssr.2023.12.015

**Published:** 2024-01-19

**Authors:** Jovan Vujic, Aljaz Hojski, Sandrine V.C. Dackam, Helga Bachmann, Didier Lardinois

**Affiliations:** 1 Department of Thoracic Surgery, University Hospital Basel, Basel, Switzerland

## Abstract

**Background:**

Sternoclavicular joint infection is rare. Operation is the treatment of choice, but there is no generally accepted approach. This report evaluated the clinical and functional results after extended surgical treatment.

**Methods:**

This single-center cohort study included 14 patients. Extended operation consisted of initial débridement with removal of the joint capsule; partial resection of the ipsilateral manubrium sterni, of the medial part of the clavicle, and sometimes of the first rib; and vacuum-assisted closure dressing. The procedure was repeated until the microbiologic findings and surgical site showed healing. Analysis of the risk factors, complications, and recurrence rate was performed. Functional results were assessed by the shortened version of the standardized Disabilities of the Arm, Shoulder, and Hand (QuickDASH) questionnaire.

**Results:**

Only 4 of 14 patients (29%) had fever and elevated infectious parameters at diagnosis. *Staphylococcus* was the most frequently observed microorganism. Grade ≥III complications according to the Clavien-Dindo classification were observed in 5 of 14 (36%) patients. Recurrence was observed in 1 patient diagnosed 2 months after hospital discharge. Clinical and functional assessment after a mean follow-up of 48 months revealed excellent results without instability of the shoulder girdle, residual pain, or functional impairment. The mean QuickDASH score in our population was 4.5 of 100 points.

**Conclusions:**

Extended surgical treatment of sternoclavicular joint infection in conjunction with assisted wound healing led to satisfying clinical and functional results.

**In Short**

▪ Extended surgical treatment of sternoclavicular joint infections is safe.
▪ There are no negative influences on upper limb function.
▪ The recurrence rate is low and patient satisfaction is high.

Sternoclavicular joint (SCJ) infection is a rare entity that is often challenging to diagnose because of its nonspecific symptoms. Most SCJ infections are caused by SCJ arthritis or sternoclavicular osteomyelitis without any traumatic events.^1^^,^^2^ Surgical treatment is the most commonly proposed therapy option.^1^^,^^3^^,^^4^ There are still controversial opinions about the optimal extent of surgical resection to achieve definitive healing.^2^, ^3^, ^4^ The literature shows that small incisions and simple débridement are associated with high recurrence rates.^3^, ^4^, ^5^ On the basis of these findings, removal of the joint capsule with resection of a part of the sternum and of the clavicle with packing and secondary closure has been proposed as a standard surgical approach.^2^, ^3^, ^4^

In this report, we present the functional results in a consecutive series of patients after more aggressive and radical surgical treatment.

## Patients and Methods

### Study Design

This single-center cohort study included 14 patients treated between May 31, 2011, and February 28, 2022. Ethical approval was received on January 18, 2022, with Registration No. 2021-02343. Eleven patients were scheduled for a prospective follow-up reevaluation to assess the mobility and function of the arm, shoulder, and hand by the shortened version of the standardized Disabilities of the Arm, Shoulder, and Hand (QuickDASH) questionnaire.^6^

### Surgical Procedure and Postoperative Treatment

Extended SCJ resection consisted of complete removal of the joint capsule, resection of a part of the manubrium sterni, and resection of the head of the clavicle and sometimes of the first sternocostal joint as well as of the first rib by using an oscillating orthopedic saw, chisel, and hammer. In the first step, a necrosectomy of infected bone parts and soft tissue was performed. Tissue samples were harvested and sent for histologic and microbiologic analysis, and a vacuum-assisted closure (VAC) dressing was applied.^7^ According to international recommendations, the VAC dressing was changed in the operating room every 3 to 4 days. The precise time interval between the reoperations depended on logistic aspects because reoperations were never performed during weekends. Changing the VAC dressing was always performed in the operating room as a sterile environment and general anesthesia were needed for repeated débridement. The decision to definitively close the wound was based on the microbiologic findings (absence of pathologic germs) and the appearance of the wound (well-vascularized granulation tissue).

In the final step, soft tissue coverage of the remnant defect was performed with a nonrotated pectoralis major muscle advancement flap. This coverage of the residual defect with muscle was routinely performed by the thoracic surgeons, not only for cosmetic purposes but mainly to bring well-vascularized tissue to fill out the rest of the tissue defect after repeated débridement.

### Retrospective Data Analysis

Descriptive analysis of several parameters collected from the patients’ clinical files was performed. These parameters included predisposing factors for SCJ infection, symptoms at hospital admission, resected structures at first operation, preoperative and postoperative inflammatory laboratory values, and morbidity according to the Clavien-Dindo classification. The type and duration of antibiotic treatment were also analyzed.

### Prospective Data Collection

Functional outcome assessment was performed using the QuickDASH questionnaire, derived from the DASH questionnaire initially published in 1996 to address concerns about shoulder and upper extremity function and stability.^6^ The score ranges from 0 (no disability) to 100 (most severe disability), and response options are presented as 5-point scales.

## Results

### Patient Characteristics

Fourteen patients with a mean age of 53 years (range, 28-70 years) were included in this report; 36% of the patients had no risk factors for infection at admission (Table 1). The mean duration of SCJ infection symptoms before initial hospital admission was 77 days; 86% of the patients presented with motion-dependent pain in the upper extremities, and 93% presented with local tenderness. Approximately half of the patients (57%) had erythema of the skin in the affected SCJ area (Table 2). Only 4 patients had fever on admission and elevated laboratory values for C-reactive protein (86% of patients), leukocytes (43% of patients), and platelets (7% of patients). The Charlson Comorbidity Index score at hospital admission was low, with a mean of 1.7.

**Table 1:** Characteristics of Patients and Conditions Predisposing to SCJ Infection

Variable	No. (%)
Demographics	
Total No. of patients included	14
Sex, female/male	2 (14)/12 (86)
Age, y, mean (range)	53 (28-70)
Risk factors in medical history	
Diabetes mellitus (with or without medication)	3 (21)
IV drug abuse (former and active)	2 (14)
Endocarditis	1 (7)
Hepatitis	3 (21)
Known rheumatic disease	2 (14)
Solid tumor or hematologic malignant neoplasm	2 (14)
Steroid therapy	3 (21)
Suppression of the immune system	3 (21)
Overall occurrence of the above risk factors in patients	9 (64)

IV, intravenous; SCJ, sternoclavicular joint.

**Table 2:** SCJ Symptoms at First Hospital Admission and Types of Resected Structures

Variable	No. (%)
SCJ symptoms at first hospital admission	
Pain (when moving the arm)	12 (86)
Local tenderness	13 (93)
Local erythema	8 (57)
Local warmness	4 (29)
Local swelling	11 (79)
Types of resected structures without revision surgeries	
SCJ	14 (100)
Clavicle	12 (86)
Manubrium sterni	12 (86)
First rib	8 (57)
Second rib	1 (7)
Sternocostal joint	8 (57)

SCJ, sternoclavicular joint.

### Extended SCJ Resection and Postoperative Treatment

The manubrium sterni and clavicle were partially resected in 86% of patients. In 57% of patients, partial (medial) resection of the first rib was additionally performed. This subgroup of patients consisted of the patients with the largest tissue defects after repeated débridement (Table 2). Overall, 12 patients underwent repeated VAC device applications (average of 2.6 dressing changes), and 12 had coverage with a muscle flap. Only in 2 patients was coverage not necessary because no residual defect was observed after repeated VAC device applications. In these 2 patients, only a limited resection of the SCJ was performed without any extension to the manubrium or to the clavicle. Interestingly, histologic results showed active osteomyelitis in 57% of the patients and acute or chronic arthritis in 93% of the patients.

Analysis of the microbiologic data revealed that *Staphylococcus* (*S. aureus* 14% and *S. epidermidis* 50%) and *Cutibacterium acnes* (43%) were most frequently detected in the tissue samples. The most often used antibiotics were amoxicillin/clavulanic acid and flucloxacillin. Antibiotic therapy was continued after hospital discharge for a mean period of 32 days.

Grade ≥III complications according to the Clavien-Dindo classification were found in 5 of 14 (36%) patients. They consisted of 2 cases of delirium (grade IIIa), 1 case of wound hematoma, 1 case of pleural effusion, 1 case of lung injury with pneumothorax (grade IIIb), and 1 case of exacerbation of left ventricular systolic dysfunction (grade IVa).

After a mean follow-up time of 48 months, recurrence of the disease was observed in 1 patient (7%) after 2 months. This patient had a well-known chronic drug consumption, and the possibility that the new SCJ infection was secondary to a local injection could not be excluded. The patient underwent reoperation with several débridement procedures with VAC device applications, and recovery was uneventful.

### Prospective Evaluation of Functional Outcome

The mean time from discharge to the study follow-up control visit was 48 months (range, 2-103 months; SD, 36 months). All patients examined reported satisfaction with functional and cosmetic outcomes after operation. All patients reported being able to perform regular work and everyday activities without difficulties. The average pain score was 0.7 on a numerical rating scale ranging from 0 (no pain) to 10 (worst possible pain). None of the patients revealed functional impairment at examination. Eleven patients were evaluated by the QuickDASH questionnaire (Table 3). The average score for the patient population was 4.5 of 100 points (SD, 5 points). The Eastern Cooperative Oncology Group performance status was rated as normal unrestricted activity (0) in 82% of patients.

**Table 3:** QuickDASH Score Results

Items	Mean per Questionnaire Item	Patient	QuickDASH Score Mean
1. Jar opening	1.0	3	0.0
2. Heavy household tasks	1.1	4	6.8
3. Carrying shopping bags	1.3	5	4.5
4. Washing the back	1.5	6	0.0
5. Cutting food	1.0	7	0.0
6. Recreational activities	1.3	8	2.3
7. Social activities	1.0	9	0.0
8. Work/regular activities	1.0	10	18.2
9. Pain	1.6	11	4.5
10. Tingling	1.1	12	9.1
11. Sleep	1.2	13	4.5
Total QuickDASH score	4.5		
Total patients	11		

QuickDASH, shortened version of the Disabilities of the Arm, Shoulder, and Hand questionnaire.

## Comment

There is still a lack of evidence and consensus on standardized diagnostics and therapy for SCJ infections.^1^^,^^3^^,^^8^ Ross and Shamsuddin^2^ reported that 24% of their patients were initially managed with simple débridement; however, at recurrence, they had to undergo extended surgical treatment. Nusselt and coworkers^1^ reported performing a simple incision with débridement and capsule removal with joint resection in fewer cases. However, complete joint removal and resection of the manubrium and clavicle were considered only if deeper structures were infected. In our experience, the extent of the resection to achieve healing cannot be accurately assessed at the first intervention. Repeated débridement with tissue samples and microbiologic analysis are needed. Our general approach is to perform extended joint resection on all patients who can tolerate surgical intervention, similar to Song and colleagues^3^ and Kachala and colleagues.^4^ In contrast to Kachala and colleagues,^4^ delayed wound closure with VAC dressing therapy is routinely added. VAC therapy has many advantages by continuously eliminating necrotic tissue and accelerating the granulation process. It also helps to reduce the size of the defect obtained after extended resection. Reoperations with repeated débridement, changing of the VAC dressings, and harvesting of tissue samples were systematically performed as part of a standard procedure. As mentioned in the Results section, an average of 2.6 reoperations was necessary to obtain samples without pathogenic organisms and good granulation tissue. We believe that these reoperations were important to accelerate healing. They should not be considered a failure of initial débridement. To our knowledge and according to the current literature, VAC dressings and repeated débridement without restricting the resection area for SCJ infection is not used as standardized therapy. At the end of the procedure, coverage of the residual tissue defect was performed with the pectoralis muscle. We consider this coverage an important step to facilitate definitive healing and to avoid recurrence of infection.

Only 4 patients had fever on admission, elevated C-reactive protein levels, and elevated leukocyte counts. This is in line with the findings of Nusselt and coworkers^1^ and Ross and Shamsuddin,^2^ who also found fever in only a few patients. One explanation could be the preoperative pain management with paracetamol and nonsteroidal anti-inflammatory drugs as well as the use of antibiotics.

The morbidity rate of 36% may appear high, but only 1 patient (7%) had a complication due to the procedure itself (wound hematoma). The other complications were rather a consequence of the anesthesia (delirium in an older patient and in a patient with alcohol abuse, decompensation of poor cardiac function) and of the operation for mediastinitis.

During follow-up, 1 recurrence of the disease was diagnosed and treated with the same surgical treatment concept. It was not possible to differentiate between a true recurrence and a second infection because of the injection of drugs in a patient with multiple recurrent abscesses in several parts of the body.

The average QuickDASH score of 4.5 points was better than that previously reported; Ayekoloye and coworkers^9^ reported a score of 10.9, and Kachala and colleagues^4^ reported a score of 19. As in our report, both authors found no important functional impairment. Our mean time of follow-up to the control visit was longer (48 vs 35 months). The highest QuickDASH score (18.2) was found for a patient who was a chauffeur by profession. This probably strongly influenced the responses in the questionnaire. However, similar to the other patients, he did not think that his upper limb function was impaired.

The excellent results on the QuickDASH questionnaire confirmed that extended operation with partial resection of the medial part of the clavicle and of the manubrium does not lead to any impairment of shoulder girdle function and has no cosmetic impact. Our findings correlate with the data of Ayekoloye and coworkers.^9^ A contributing factor to the good functional results achieved in our report could be that most of the patients underwent targeted physiotherapy during their hospital stay and after discharge.

We are aware of the limitation of the analysis due to the low number of patients included, but SCJ infections remain a rare clinical entity.

In conclusion, our report suggests that initial extended operation followed by repeated débridement and assisted wound healing by the use of VAC dressing leads to excellent control of infection, does not impair upper extremity function, and should be considered a reference treatment in patients with SCJ infection.
